# *Ixodes ricinus *ticks are reservoir hosts for *Rickettsia helvetica *and potentially carry flea-borne *Rickettsia species*

**DOI:** 10.1186/1756-3305-2-41

**Published:** 2009-09-04

**Authors:** Hein Sprong, Peter R Wielinga, Manoj Fonville, Chantal Reusken, Afke H Brandenburg, Fred Borgsteede, Cor Gaasenbeek, Joke WB van der Giessen

**Affiliations:** 1 Laboratory for Zoonoses and Environmental Microbiology, National Institute for Public Health and Environment (RIVM), Antonie van Leeuwenhoeklaan 9, P.O. Box 1, Bilthoven, the Netherlands; 2 Department of Medical Microbiology, Public Health Laboratory, Leeuwarden, the Netherlands; 3 Central Veterinary Institute of Wageningen University, Lelystad, the Netherlands

## Abstract

**Background:**

Hard ticks have been identified as important vectors of rickettsiae causing the spotted fever syndrome. Tick-borne rickettsiae are considered to be emerging, but only limited data are available about their presence in Western Europe, their natural life cycle and their reservoir hosts. *Ixodes ricinus*, the most prevalent tick species, were collected and tested from different vegetation types and from potential reservoir hosts. In one biotope area, the annual and seasonal variability of rickettsiae infections of the different tick stages were determined for 9 years.

**Results:**

The DNA of the human pathogen *R. conorii *as well as *R. helvetica, R. sp. IRS *and *R. bellii-like *were found. Unexpectedly, the DNA of the highly pathogenic *R. typhi *and *R. prowazekii *and 4 other uncharacterized *Rickettsia spp*. related to the typhus group were also detected in *I. ricinus*. The presence of *R. helvetica *in fleas isolated from small rodents supported our hypothesis that cross-infection can occur under natural conditions, since *R. typhi/prowazekii *and *R. helvetica *as well as their vectors share rodents as reservoir hosts. In one biotope, the infection rate with *R. helvetica *was ~66% for 9 years, and was comparable between larvae, nymphs, and adults. Larvae caught by flagging generally have not yet taken a blood meal from a vertebrate host. The simplest explanation for the comparable prevalence of *R. helvetica *between the defined tick stages is, that *R. helvetica *is vertically transmitted through the next generation with high efficiency. The DNA of *R. helvetica *was also present in whole blood from mice, deer and wild boar.

**Conclusion:**

Besides *R. helvetica*, unexpected rickettsiae are found in *I. ricinus *ticks. We propose that *I. ricinus *is a major reservoir host for *R. helvetica*, and that vertebrate hosts play important roles in the further geographical dispersion of rickettsiae.

## Background

The majority of emerging infectious diseases originates from wildlife instead of companion animals [1], and more than half is caused by bacteria and rickettsiae [2]. Rickettsiae are fastidious, mostly obligate intracellular alpha-proteobacteria. They have a worldwide distribution and are the causative agents of severe human infections. Rickettsiae are usually not directly transmissible from human to human [3], but are transmitted via arthropod vectors such as ticks, mites and blood sucking insects, which transmit rickettsiae between humans but also from animals to humans [4]. The rat flea is the main vector of *R. typhi*, which causes murine typhus and *R. prowazekii *is transmitted between humans by fleas and lice and is the causative agent of epidemic typhus [5]. Hard ticks (*Ixodidae*) have been identified as vectors of the spotted fever syndrome in humans, which is caused by at least 15 different *Rickettsia *species. Two infamous members of this group are *R. rickettsii*, the causative agent of Rocky Mountain spotted fever and *R. conorii*, the causative agent of Mediterranean spotted fever [6].

The geographic distribution of ticks and the rickettsiae they transmit depends on environmental factors like climate, biotope and on the abundance of ticks and their hosts. However, the impact of human behaviour on the environment, together with a changing climate undoubtedly affects the spread of ticks and the pathogens they carry [2,7,8]. In particular, rapidly evolving rickettsiae may adapt to different hosts and environmental conditions, and prevalence of currently minor pathogenic species may be favoured by changing environmental conditions [9]. The number of representatives of the genus *Rickettsia *has increased over the last decades as a result of improved identification techniques [6]. Emerging *Rickettsia *species such as *R. helvetica*, have been isolated from ticks in various European countries such as Switzerland, Sweden, Poland, Germany, France and The Netherlands [10-16]. To date, the pathogenic potential is unclear but *R. helvetica *infection has been suspected in acute perimyocarditis, unexplained febrile illness and sarcoidosis [17-22].

In the Netherlands, *Ixodes ricinus *is the most predominant tick species. Earlier studies in the Netherlands have shown that these ticks carry different *Borrelia *spp, *Anaplasma *spp and sporadically *Babesia *species [23,24]. Studies by Dutch practitioners in the period 1994-2005 showed an apparent three-fold increase in the tick biting incidence and the incidence of Lyme disease, indicated by the diagnosis of erythema migrans [25,26]. Our long-term aims are to monitor, prevent and control tick-borne diseases. A sentinel for tick-borne diseases in humans might be the occurrence of these pathogens in ticks. Identification of key factors that affect the prevalence of tick-borne diseases will facilitate effective control measures.

In order to assess the occurrence of pathogenic tick-borne rickettsiae in The Netherlands, ticks from different vegetation types and ticks originating from humans and animals were collected and tested. In one biotope, the annual and seasonal variability of *R. helvetica *infections of the different tick stages was determined and potential reservoir hosts, such as rodents including their ticks and fleas and other wildlife were studied.

## Results

### Collection of ticks

**Figure 1:**
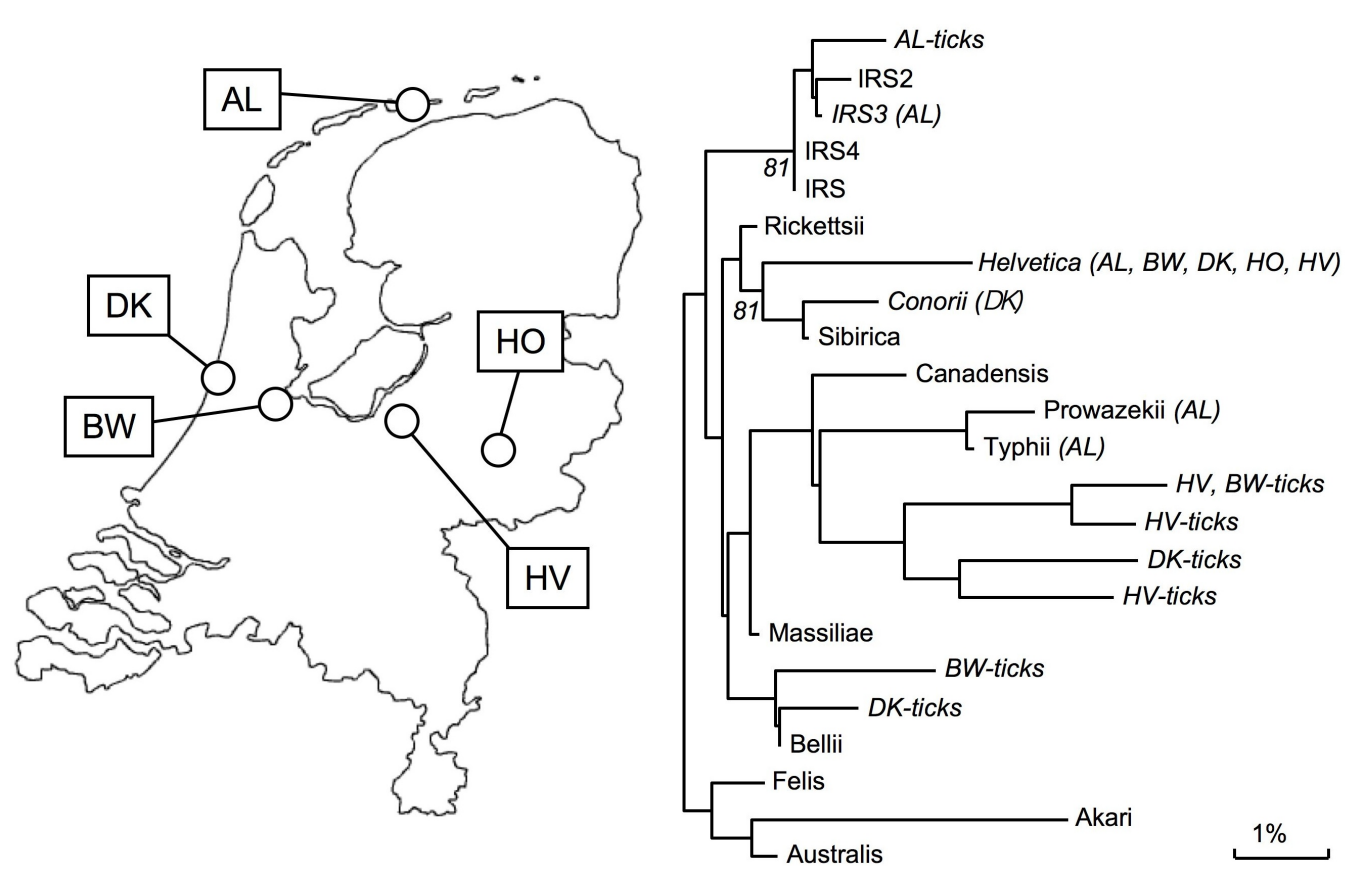
**Occurrence of Rickettsia spp. in The Netherlands**. Left: Map of the Netherlands showing the five locations that were sampled for this study. Right: Genetic variation of *Rickettsia *species found in The Netherlands. The phylogenetic relationship was based on a part of the 16SrRNA sequence: base 41 until 383 of *R. prowazekii*. The evolutionary distance values were determined by the method of Jukes and Cantor, and the tree was constructed according to the neighbor joining method. Cophenetic correlation coefficients, which are lower than 89%, are indicated at the nodes. 16SrRNA sequences from *R. SP. IRS *(L36102), *IRS2 *(DQ100164), *IRS3 *(AF141907) IRS4 (AF141908), *rickettsii *(L36217), *helvetica *(L36212), *conorii *(L36105), *sibirica *(L36218), *canadensis *(L36104), *prowazekii *(U29135), *typhi *(M20499), *massiliae *(L36106), *bellii *(L36103), *felis *(DQ102712), *akari *(L36099), and *australis *(L36101) were from Genbank. Between brackets the regions where the *Rickettsia *species were identified (AL = Ameland; BW = Bijlmerweide; DK = Duin en Kruidberg; HO= Heumesoord; HV= Houtvesterijen). No names are given to *Rickettsia *(sub)species which are not identical to the sequences in Genbank(e.g. AL-ticks).

To investigate the presence of rickettsiae and the possible emergence of *Rickettsia *species in the Dutch tick population, 1735 ticks were collected between 2000 and 2008 (Table 1) from 5 geographically different locations (Figure 1 left). Ticks from Bijlmerweide (BW), Koninklijke Houtvesterijen (HV), Duin en Kruidberg (DK), and Heumesoord (HO) were collected by blanket dragging [27]. BW is a typical city park near Amsterdam with many deciduous trees and a few shrubs with a rich secondary vegetation. HV is an oak forest where ninety percent of the area is covered with blueberries. DK is a dune area rich in vegetation were several species of deciduous trees and shrubs were present, and 60% of the soil was covered with vegetation litter. HO is a typical heather area which consisted of heather only, with a single pine tree and very little vegetation litter. Only very scarce and incomplete information is available about the fauna present in these areas. Ticks from Ameland (AL) originated from humans who were bitten by ticks on the isle in the Wadden Sea, and who had gone to the local general practitioner for removal of the tick [28].

**Table 1:** Prevalence of *R. helvetica *in ticks.

**Location**	**Area type**	**Collection (Years)**	**Ticks (n) *all 3 stages***	***R. helvetica***
Ameland (AL)	Vegetation rich dune	Tick bites (2004-06)	169	12%
Bijlmerweide (BW)	City park	Flagging (2000-02)	39	36%
Duin en kruidberg (DK)	Vegetation-rich dune	Flagging (2000-08)	1295	66%
Heumesoord (HO)	Heather/forest	Flagging (2007)	89	21%
Houtvesterijen (HV)	Forest	Flagging (2000-02)	143	6%

Ticks from different geographical locations in The Netherlands were tested for the presence of *R. helvetica*. Ticks (larvae, nymphs and adults) were collected from different area types, in different years, and the infection rate with *R. helvetica *is expressed as a percentage. As the infection rates between the different tick stages were not significantly different within each location, the overall infection rate is presented.

### Detection of rickettsiae in arthropods

Total DNA from ticks was extracted and amplified by PCR using generic primers against the 16S rRNA gene of all *Rickettsia *species [27]. PCR products were analyzed by RLB. *Rickettsia *isolates, which only reacted with the catch all probe on RLB, were identified by sequencing the 16S rRNA PCR product and characterized by phylogenetic analysis (Figure 1 right). Forty-one percent of all ticks (719 of 1735) were infected with *R. helvetica*, varying from only 6% in a blueberry-rich oak forest (HV) to 66% in a typical vegetation-rich dune area (DK; Table 1). The PCR products of 16S rRNA of 15 *R. helvetica *field isolates were sequenced and the 360 basepair-region showed no sequence variation with the corresponding sequence in Genbank (L36212). Also, PCR amplification and sequencing part of the citrate synthase gene [29] of *R. helvetica *from different locations yielded identical sequences to the corresponding sequences deposited in Genbank (U59723: not shown). In Ameland (AL), 4.7% of ticks were infected with *R. sp. IRS *[27]. Their sequences were identical or almost identical (>99%) to published sequences [29] (Figure 1). Remarkably, in 2.4% of the nymphs from Ameland, which originated from human subjects, *R. typhi *and *R. prowazekii *was detected. This finding was unexpected, since these pathogens almost exclusively occur in fleas and/or lice [30,31]. The 360 basepair-region of the 16S rRNA DNA sequences of *R. typhi *and *R. prowazekii *found in tick lysates were identical to the sequences submitted to Genbank accession numbers M20499 and U29135, respectively. We are not aware of any organism that has exactly the same sequence as *R. typhi *or *R. prowazekii *in this region. The closest match to our sequences is *R. massiliae *which has 7 mismatches with the sequence of *R. typhi*. PCR amplifications of a part of the citrate synthase of *R. typhi *and *R. prowazekii *using two different primer sets in these tick lysates were negative (not shown). Here, all the *R. typhi *and *R. prowazekii *isolates were found in ticks which fed on human and were from one location (AL). In two recent field studies carried out in the Netherlands, we have also found both *R. typhi *and *R. prowazekii *in unfed ticks caught by blanket-dragging on 4 other locations (not shown).

One straightforward explanation for the detection of *R. typhi *and *R. prowazekii *is that ticks have obtained these organisms via a blood meal from an infected rodent. Conversely, fleas might obtain *Rickettsia *species, which are normally associated with ticks, via a blood meal on an infected rodent. In order to verify this possibility, we tested 24 fleas isolated from *Myodes glareolus *(bank vole) and *Apodemus sylvaticus *(yellow-necked field mouse) for the presence of rickettsiae. Three samples from fleas reacted positive in the RLB, and two of these samples were identified as *R. helvetica *by sequencing (not shown).

Contamination of tick lysates or PCR reactions with the plasmid controls of *R. typhi, R. prowazekii *and *R. bellii *was excluded: these two plasmids could also be detected using M13 forward and reverse primers up to 1fg plasmid DNA per sample. PCR amplifications on all the *R. typhi*- and *R. prowazekii*-positive tick lysates with M13 primers were all negative (not shown). Furthermore, In DK and BW, a *Rickettsia *species (<1%) was found to be similar, but not identical to *R. bellii *(Figure 1). In DK and HV, *Rickettsia *species were identified that were related to but different from *R. typhi *and *R. prowazekii*.

### Detection of rickettsiae in wildlife

The infection rate of ticks with microorganisms depends on the interaction with vertebrate hosts and may vary between seasons and years [32]. To determine which wildlife may be considered as vertebrate host for rickettsiae in The Netherlands, whole blood samples of wildlife were collected between 2001 and 2008 from the different areas and tested by PCR. From the 146 mice tested with PCR 29%, 14%, and 1.4% were infected with *R. helvetica*, *R. sp. IRS*, and *R. conorii*, respectively. 19% of the roe deer and 7% of the wild boar were positive for *R. helvetica*, but not for other *Rickettsia spp *No rickettsiae were found in red deer (Table 2).

**Table 2:** Presence of rickettsiae in Dutch wildlife.

**Animal (n)**	***R. helvetica *(n)**	***R. conorii *(n)**	***R. SP. IRS *(n)**
Mouse (146)	29% (43)	1.4% (2)	14% (20)
Roe deer (21)	19% (4)	0	0
Wild boar (29)	6.9% (2)	0	0
Red deer (10)	0	0	0

Wildlife from different geographical locations (see Figure 1) was tested for the presence of Rickettsiae. Whole blood samples of animals (number) were collected between 2000 and 2002, and the infection rate with *R. helvetica *and *R. conorii *is expressed as a percentage.

### Transmission dynamics of R. helvetica

**Figure 2:**
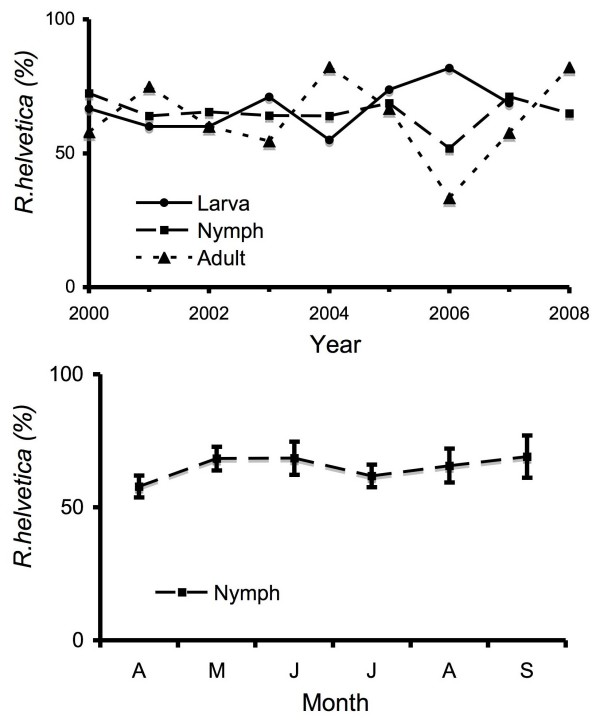
**Variability of tick infection with *R. Helvetica***. *R. helvetica*-positive ticks from DK were divided per life stage and were expressed as a percentage per year (A) or per month (B). Monthly averages and standard deviations were calculated. In figure 2A, the percentage of infected adult ticks in 2006 is not representative, because only three adult ticks were caught. In other years between 12 and 26 adults were caught and tested.

To gain insight into the transmission dynamics of the most abundant *Rickettsia *species between ticks and vertebrate hosts, the occurrence of *R. helvetica *infection in ticks was determined over time. The annual prevalence of *R. helvetica *in DK was practically constant in all three tick stages for 9 years, from 2000 until 2008 (Figure 2A). In contrast, the prevalence of *Borrelia *in nymph and adults in this area varied considerably, between 2 and 12% [23](not shown). Typing of the ticks showed that all stages (larvae, nymphs and adults) of *I. ricinus *were associated with approximately similar infection degrees of *R. helvetica *(Figure 2A). Furthermore, the monthly infection rate of nymphs did not change significantly either (Figure 2B), whereas a seasonal fluctuation is observed for *Borrelia *in nymph and adults in the same area [23](not shown). The number of larvae and adults caught were too low for analysis of the monthly variation.

## Discussion

Our investigations corroborate the wide distribution of *R. helvetica *in the Netherlands, which was also found by Nijhof *et al*. in ticks from domesticated animals [12]. The overall occurrence of *R. helvetica *in ticks is comparable with those reported from other European countries which varies between 4-16% [14,33-36], and varied between different biotopes (Table 1). In a vegetation-rich dune area (DK), an exceptionally high prevalence of *R. helvetica *was found of ~66% (Table 1). This high percentage was seen during all 9 years of this study. As far as we know, it is the highest prevalence ever reported for *R. helvetica*. How the striking differences in the percentage of *R. helvetica *infected ticks are coupled to the environmental differences is unclear, and a topic for current investigations.

The occurrence of *R. helvetica *in ticks was persistent between 2000 and 2008, and demonstrated that *R. helvetica *has established a stable life cycle in The Netherlands (Figure 2A). The infection rate with *R. helvetica *is comparable between larvae, nymphs, and adult (Figure 2A). Also, the infection rate of nymphs is hardly affected by seasons (Figure 2B). In general, *Ixodes ricinus *larvae caught by flagging have not yet had a blood meal from a vertebrate host. Therefore, the simplest explanation for the comparable prevalence of *R. helvetica *between the defined ticks stages is, that *R. helvetica *is vertically transmitted through the next generation with high efficiency. Efficient transovarial transmission has already been demonstrated for other rickettsiae and has also been demonstrated for *R. helvetica *under laboratory conditions [37,38]. Pathogens that benefit from efficient transovarial transmission hardly depend on vertebrate hosts as reservoirs, and *Ixodes ricinus *can therefore be considered as a reservoir host. *R. helvetica *DNA was also found in whole blood from mice, roe deer and wild boar. No clinical signs were reported from these animals. Here, we show that mice and roe deer may act as reservoir hosts, and could be involved in the further geographical dispersion of *R. helvetica*. We did not detect any rickettsia in red deer, though red deer are used by ticks for blood meals (not shown). This finding may imply that red deer are not good hosts for rickettsia.

The finding of the highly pathogenic *R. typhi *and *R. prowazekii *in naturally infected *I. ricinus *is unexpected, and this is thought to be the first time this is reported. However, *R. prowazekii *has been isolated sporadically from several other ixodid ticks [39-41]. In this study, the ticks with *R. typhi *and *R. prowazekii *were derived from human subjects. More recent field studies from our laboratory have found both pathogens in unfed ticks by blanket dragging and in other areas as well (not shown). The presence of *R. typhi and R. prowazekii *in *I. ricinus *ticks is plausible since *R. typhi*, *R. prowazekii*, and *R. helvetica *share mice as vertebrate hosts, and mice are infested by both ticks and fleas. We demonstrated that *R. helvetica *is found in rodent fleas as well. Ticks from three habitats contained rickettsia species related to *R. typhi/prowazekii *(Figure 1). We assume that *R. typhi *and *R. prowazekii *still circulate in (Dutch) rodents using fleas as vectors. Possibly, ticks sporadically become infected via a blood meal on an infected rodent. Experimental infections showed that *R. prowazekii *could be maintained trans-stadially, but failed to demonstrate that ticks could function as efficient vectors for *R. prowazekii *[39-41]. Until now, the presence of *R. typhi *and *R. prowazekii *in Dutch ticks has not led to reported disease cases in humans.

The other *Rickettsia *species which were found included *R. conorii, R. SP. IRS, R. bellii-like *and 4 rickettsiae related to the typhus group (Figure 1). Additionally, *R. australis *and *R. raoulti *were recently identified in The Netherlands in two other studies, also [12,13]. These findings demonstrate that *I. ricinus *ticks harbor many different *Rickettsia *species, some of which have already been shown to be an agent of human rickettsiosis. Whether *R. helvetica *exposure through tick-bites constitutes a risk to human health is unclear. The occurrence and pathogenicity of *Rickettsia *species in humans in The Netherlands has remained largely unaddressed, probably because the early signs and symptoms of rickettsial infections are notoriously non-specific, mimicking flu-like illnesses, the medical community is poorly aware of potential rickettsial diseases acquired, and powerful diagnostic tools are lacking.

In this study, only the DNA of Rickettsiae has been detected and identified in samples from wild life, ticks and fleas. In order to prove the presence of the corresponding infectious agents, their viability needs to be tested by *in vitro *culture or infection experiments of laboratory animals. Future studies aim to investigate whether these *Rickettsia *species in ticks are an emerging risk to public health.

## Materials and methods

### Collection of ticks, fleas and wildlife and extraction of DNA

Ticks were collected between 2000 and 2008 by flagging the vegetation at 4 different habitats. Ticks were immersed in 70% ethanol and stored at -20°C before testing. Every year, from April until September, a maximum of 50 questing ticks (larvae, nymphs, adults) were collected monthly in a vegetation-rich dune area, Duin en Kruidberg [23]. On the Island of Ameland ticks were collected from persons presenting with a tick bite at the local general practitioner. Based on morphological criteria, all ticks were identified as *Ixodes ricinus*. In doubtful cases, sequencing of tick mitochondrial 16S rRNA confirmed the *I. ricinus *origin [42]. DNA from ticks was extracted by alkaline lysis [23]. Fleas were collected from *Myodes glareolus *(bank vole) and *Apodemus sylvaticus *(wood mouse), trapped in the period February to July 2008. The fleas were immediately stored on dry ice and subsequently at -80°C before testing. Total DNA was extracted as follows. Fleas were disrupted using liquid N_2 _and pestles, and homogenized in 600 μl buffer RLT using a Qiashredder homogenizer according to the manufacturer's instructions (RNeasy minikit, Qiagen). DNA was extracted from 300 μl homogenate using the QIAamp DNA mini kit (Qiagen). DNA was eluted using 50 μl elution buffer. Whole blood samples from wildlife were collected from the same areas and in the same period. DNA was extracted using the QIAamp-DNA-Blood-Mini-Kit (Qiagen-Benelux, Venlo, the Netherlands) according to the manufacturer's instructions.

### Detection of *Rickettsia *spp

The presence of the DNA of different *Rickettsia *species was determined by PCR followed by reverse line blotting (RLB) as previously described. [27]. Briefly, the 16S rRNA gene was amplified by PCR with the HotStarTaq master mix (Qiagen, Venlo, The Netherlands) using 5'-AACGCTATCGGTATGCTTAACA and 5'-Biotin-ACTCACTCGGTATTGCTGGA-3 as primers with the following conditions: 15 min 94°C, then cycles of 20 s 94°C, 30 s 67°C, 30 s 72°C lowering the annealing temperature 1°C each cycle till reaching 67°C, then 40 cycles at this annealing temperature and ending by 10 min 72°C. For RLB analysis of the PCR product the following amino labeled probes were used: 5'-TTTAGAAATAAAAGCTAATACCG (catch all), CTTGCTCCAGTTAGTTAGT (*R. conorii*), GCTAATACCATATATTCTCTATG (*R. helvetica*), and GTATATTCTCTACGGAAAAAAG (*R. SP. IRS3*). Positive controls were plasmids containing the 16S rRNA sequence from *R. helvetica, R. conorii, R. typhi *and *R. prowazekii *and were kindly provided by Leo Schouls [27]. To minimize cross contamination and false-positive results, positive and negative controls were included in each batch tested by the PCR and RLB assays. In addition, DNA extraction, PCR mix preparation, sample addition, and PCR analysis were performed in separated, dedicated labs. PCR amplicons were purified with the Qiaquick gel extraction kit (Qiagen Inc.) and sequenced using an ABI PRISM BigDye Terminator Cycle sequencing Ready Reaction kit (Perkin Elmer, Applied Biosystems). All sequences were confirmed by sequencing both strands, and unique sequences were submitted to Genbank (GQ849209-GQ849220). Sequences were compared with sequences in Genbank using BLAST. Phylogenetic analysis was performed using Bionumerics version 5.1 (Applied Maths, Gent, Belgium). PCR amplification of parts of the citrate synthase gene was done exactly as described by De Sousa and colleagues, using cs409d/rp1258n and cs535d/Rp1258 as primer pairs [29].

## Abbreviations

AL: Ameland; BW: Bijlmerweide; DK: Duin en Kruidberg; HV: Koninklijke Houtvesterijen; HO: Heumesoord; RLB: reverse line blot.

## Competing interests

The authors declare that they have no competing interests.

## Authors' contributions

HS performed (bioinformatics) analyses, wrote the final manuscript. PRW analysed data, wrote the initial draft. MF collected data, developed new methodology. CR was responsible for the collection and analysis of the flea data. AHB was involved in the collection of ticks from humans. FB designed the study, acquired funding. CG conducted most of the field work, was involved in the study design JWBG designed the study, acquired funding and conducted data analysis. All authors approved the final version.
